# Emerging henipaviruses and future pandemic preparedness: diagnostic delays, zoonotic spillover, and One Health challenges

**DOI:** 10.3389/fpubh.2026.1875406

**Published:** 2026-10-12

**Authors:** Abdeldjalil Zitouni

**Affiliations:** Private Dental Practice, Constantine, Algeria

**Keywords:** Hendra virus, henipavirus, Langya virus, Nipah virus, pandemic preparedness, zoonotic spillover

## Abstract

Henipaviruses are among the most concerning emerging zoonotic pathogens because of their high mortality rates, neurological involvement, broad host range, and outbreak potential. This mini review discusses diagnostic limitations, outbreak amplification, zoonotic spillover, surveillance fragmentation, and future pandemic preparedness strategies associated with Nipah, Hendra, and Langya henipaviruses. Unlike previous reviews mainly focused on descriptive virology and epidemiology, this review emphasizes diagnostic preparedness, surveillance weaknesses, outbreak amplification mechanisms, and future One Health response strategies. Current evidence suggests that delayed diagnosis, fragmented surveillance systems, environmental disruption, and limited rapid diagnostic capacities continue to increase future spillover risks. Strengthening One Health preparedness frameworks, rapid diagnostics, genomic surveillance, and international collaboration remains essential for reducing future pandemic threats.

## 1 Introduction

Emerging zoonotic viruses continue to represent a major threat to global public health (1, 2). Henipaviruses have attracted increasing scientific attention because of their elevated mortality rates, neurotropic properties, and epidemic potential (3, 4). Fruit bats of the Pteropus genus are recognized as the primary reservoirs for Nipah and Hendra viruses, while additional unidentified reservoirs may exist for other emerging henipaviruses (3, 4).

More recently, the identification of Langya henipavirus in China renewed concerns regarding future zoonotic spillover events and underrecognized transmission pathways (5, 6).

Unlike previous narrative reviews mainly focused on virology and descriptive epidemiology, the present mini review specifically emphasizes diagnostic preparedness, surveillance fragmentation, outbreak amplification mechanisms, and future pandemic-response vulnerabilities associated with emerging henipaviruses.

## 2 Emerging henipaviruses

Nipah virus remains the most concerning henipavirus because of mortality rates reaching 40–75% and documented human-to-human transmission in Bangladesh and India (3, 4). Hendra virus continues to represent an important zoonotic threat in Australia despite the availability of veterinary vaccines (3).

Langya henipavirus remains less characterized but highlights the continuing emergence of novel zoonotic henipaviruses capable of infecting humans (5, 6). The discovery of Langya henipavirus also suggests that additional unidentified henipaviruses may already be circulating in animal reservoirs with limited human surveillance (5, 6). This possibility reinforces concerns regarding future zoonotic emergence events and underrecognized spillover transmission (6, 7).

## 3 Diagnostic and surveillance challenges

A major limitation identified across multiple henipavirus outbreaks is the persistent delay between symptom onset and definitive laboratory confirmation. Clinical manifestations frequently overlap with influenza, pneumonia, and viral encephalitis, increasing the probability of early misdiagnosis (4, 8) (Table 1).

**Table 1:** Comparison of major emerging henipaviruses.

Virus	Reservoir	Mortality	Human-to-human transmission	Geographic region	Availability of vaccine	Major challenges
Nipah virus	Fruit bats	40–75%	Limited but documented	Malaysia, Bangladesh, India	Experimental only	High mortality, delayed diagnosis
Hendra virus	Fruit bats	~57%	Not confirmed	Australia	Veterinary vaccine available	Animal spillover surveillance
Langya virus	Possibly shrews	Unknown/limited data	Not confirmed	China	None	Limited epidemiological data

Diagnostic delay not only affects individual patient outcomes but may also significantly amplify outbreak propagation by delaying isolation measures, contact tracing, and public health responses (2, 4).

Another important challenge involves biosafety constraints. Confirmatory laboratory testing frequently requires high-containment laboratory facilities that remain unavailable in many high-risk regions (1, 8).

Recent advances in portable RT-PCR systems, CRISPR-based diagnostics, and field-deployable biosensors may offer promising future solutions. However, these technologies remain insufficiently implemented in endemic regions (8).

The persistence of fragmented surveillance systems also represents a critical weakness in current preparedness strategies. Human, veterinary, wildlife, and environmental surveillance frequently operate independently rather than through integrated One Health frameworks (1, 9).

## 4 Outbreak amplification and public health risks

The amplification potential of henipavirus outbreaks extends beyond direct viral transmission alone. Multiple ecological, healthcare, socioeconomic, and environmental factors may collectively increase outbreak severity (7, 10).

Outbreaks occurring in densely populated regions with limited healthcare infrastructure may rapidly overwhelm local systems. Modern transportation networks may theoretically facilitate cross-border dissemination before diagnosis (1, 2).

Environmental disruption, climate change, deforestation, and increasing human encroachment into wildlife habitats may progressively increase zoonotic spillover frequency in the coming decades (7, 10).

## 5 Future preparedness and One Health strategies

The COVID-19 pandemic highlighted the importance of proactive preparedness rather than reactive crisis management. Preparedness strategies should include expansion of genomic surveillance networks, strengthening laboratory capacities, rapid outbreak reporting systems, development of portable diagnostics, and multidisciplinary One Health surveillance approaches (1, 2, 9).

Artificial intelligence-assisted surveillance systems, wastewater epidemiology, genomic monitoring, and digital outbreak prediction tools may become increasingly important for identifying early spillover signals and monitoring emerging zoonotic threats (1, 2).

Artificial intelligence and digital epidemiology tools may also contribute to outbreak prediction and surveillance optimization. Public communication strategies remain essential because misinformation and delayed reporting may complicate outbreak management (2).

Investment in vaccine research also remains critical. Although promising preclinical vaccine candidates and monoclonal antibodies have been reported, no widely available human vaccine currently exists for Nipah virus (1, 4).

## 6 Future directions

Future studies should prioritize integrated genomic surveillance, portable diagnostic development, ecological monitoring of wildlife reservoirs, and predictive modeling of zoonotic spillover risks (1, 7, 8, 10). International preparedness frameworks should also incorporate rapid data-sharing mechanisms and coordinated One Health response systems (1, 2, 9).

## 7 Limitations

This mini review has several limitations. First, the number of available studies specifically focusing on newly identified henipaviruses such as Langya virus remains relatively limited (5, 6). Second, much of the available evidence originates from observational reports and narrative reviews rather than large prospective studies. Finally, the rapidly evolving nature of emerging zoonotic diseases may lead to continuous changes in epidemiological knowledge and preparedness strategies.

## 8 Conclusion

Emerging henipaviruses represent a continuing threat to global public health because of their zoonotic origin, elevated mortality rates, and outbreak potential. Strengthening One Health surveillance systems, improving rapid diagnostic capacities, expanding international collaboration, and investing in preparedness strategies are essential to reduce future risks (1, 2, 8, 9).

Continuous monitoring of emerging henipaviruses should remain a global public health priority (1, 2).

## References

[1] World Health Organization. WHO Research and Development Blueprint for Priority Pathogens. Geneva: World Health Organization (2024).

[2] Centers for Disease Control and Prevention. CDC Emerging Zoonotic Viral Threats and Pandemic Preparedness Report. Atlanta, GA, USA: Centers for Disease Control and Prevention (2024).

[3] Chua KB Bellini WJ Rota PA Harcourt BH Tamin A Lam SK . Nipah virus: a recently emergent deadly paramyxovirus. Science. (2000) 288:1432–5. doi: 10.1126/science.288.5470.1432, 10827955

[4] Luby SP. The pandemic potential of Nipah virus. Antivir Res. (2013) 100:38–43. doi: 10.1016/j.antiviral.2013.07.011, 23911335

[5] Zhang XA Li H Jiang FC Zhu F Zhang YF Chen JJ . A zoonotic henipavirus in febrile patients in China. N Engl J Med. (2022) 387:470–2. doi: 10.1056/NEJMc2202705 35921459

[6] Shafaati M Zandi M. Langya henipavirus (LayV) as an emerging zoonotic disease: a mini-review. New Microbes New Infect. (2025) 68:101643. doi: 10.1016/j.nmni.2025.101643, 41141920 PMC12547270

[7] Plowright RK Ahmed AN Coulson T Crowther TW Ejotre I Faust CL . Zoonotic spillover pathways and future pandemic prevention strategies. Lancet Planet Health. (2024) 15:2577. doi: 10.1038/s41467-024-46151-9

[8] Mazzola LT Kelly-Cirino C. Advances in rapid diagnostics for emerging zoonotic pathogens. Nat Rev Microbiol. (2025) 4:e001118. doi: 10.1136/bmjgh-2018-001118

[9] One Health High-Level Expert Panel. One health joint action framework. Lancet. (2025) 401:530–3. doi: 10.1016/S0140-6736(23)00086-7 PMC9851626 36682373

[10] Carlson CJ Albery GF Merow C Trisos CH Zipfel CM Eskew EA . Climate change increases cross-species viral transmission risk. Nature. (2022) 607:555–62. doi: 10.1038/s41586-022-04788-w, 35483403

